# Mystics at war: Padre Pio and Ludwig Wittgenstein

**DOI:** 10.1515/sem-2025-0091

**Published:** 2025-06-04

**Authors:** Francesco Galofaro

**Affiliations:** 18983 Università IULM, Milan, Italy

**Keywords:** semiotic labor, speech-acts, metasemiotics, experience, semio-technics, travail sémiotique, actes de langage, métasémiotique, expérience, sémio-technique

## Abstract

Max Weber describes a coherent ideal type of mystic, characterized by passivity and “living on berries in the woods, or on alms.” This way, Max Weber disregards the paradoxicality of mystical discourse, selecting a coherent path in a contradictory semantic universe and producing a semiotic ideology, functional to his argument about the relation between capitalism and Protestantism. On the contrary, mystics operate inside the social world and take sides in its conflicts. They react to social crises, such as war, by linking a spiritual reading to their bodily experiences. Eco’s notion of “semiotic labor” can be useful to analyze how this semiotic relation is produced in mystical writings through metasemiotic statements. The paper focuses on two case-studies: Padre Pio’s letters and Ludwig Wittgenstein’s diaries, both written during World War I. The analysis will highlight a common structure: both mystics associate spiritual values with pain, anguish, and fear through catalysis, interpreting them as divine trials. This is done thanks to metasemiotic assertions introduced and validated by speech acts. This structure is interpreted as a semio-technique, producing the semantic values with which the subject wishes to join.

## Résumé

Max Weber décrit un type idéal cohérent de mystique, caractérisé par la passivité et «vivant de baies dans les bois ou d’aumônes». De cette façon, Max Weber ne tient pas compte de la paradoxalité du discours mystique, qui choisit une voie cohérente dans un univers sémantique contradictoire et produit une idéologie sémiotique, voie utile à son argument sur la relation entre le capitalisme et le protestantisme. Au contraire, les mystiques opèrent à l’intérieur du monde social et prennent parti dans ses conflits. Ils réagissent aux crises sociales, telles que la guerre, en liant une lecture spirituelle à leurs expériences corporelles. La notion de «travail sémiotique» d’Umberto Eco peut être utile pour analyser la manière dont cette relation sémiotique est produite dans les écrits mystiques par le biais d’énoncés métasémiotiques. L’article se concentre sur deux études de cas: les lettres de Padre Pio et les journaux intimes de Ludwig Wittgenstein, tous deux écrits pendant la Première Guerre mondiale. L’analyse mettra en évidence une structure commune: les deux mystiques associent des valeurs spirituelles à la douleur, à l’angoisse et à la peur par le biais de la catalyse, en les interprétant comme des épreuves divines. Cela se fait grâce à des assertions métasémiotiques introduites et validées par des actes de langage. Cette structure est interprétée comme une sémio-technique, produisant les valeurs sémantiques auxquelles le sujet souhaite s’associer.

## 1 Introduction: mysticism and political engagement

The relationship between mystics and politics illustrates the difference between specific semiotics and sociological approaches regarding methods and conclusions. In particular, textual semiotics aims for a faithful analysis of empirical data restricting and discussing extrapolations outside the considered corpus, up to the limit of a *mathesis singularis* (Barthes 1980). On the contrary, a sociological tradition started by Max Weber reconstructs ideal types, often renouncing accuracy in the description of individual cases.1This project has received funding from the European Research Council (ERC) under the European Union’s Horizon 2020 research and innovation programme (grant agreement No. 757314).


Weber (1978 [1921]: 541–544) opposes mysticism and asceticism as two ideal types of religious way to redemption. Weber’s opposition bases on a homology:

Asceticism/Mysticism=Active/Passive

Weber further distinguishes between world-rejecting and inner-worldly asceticism: the latter refers to Protestant radical denominations, whose ethics would ground the capitalist deontology. According to protestant asceticism, vocation and profession coincide, as suggested by the German word *Beruf*.

Weber describes alternative points of view of the mystic and of the ascetic:

From the standpoint of a contemplative mystic, the ascetic appears, by virtue of his transcendental self-maceration and struggles, and especially by virtue of his ascetically rationalized conduct within the world, to be forever involved in all the burdens of created things, confronting insoluble tensions between violence and generosity, between matter-of-factness and love. (Weber 1978 [1921]: 546)

[F]rom the converse standpoint, the contemplative mystic appears not to be thinking of God, the enhancement of his kingdom and glory, or the fulfilment of his will, but rather to be thinking exclusively about himself. Therefore, the mystic lives in everlasting inconsistency, since by reason of the very fact that he is alive he must inevitably provide for the maintenance of his own life. (Weber [1978] 1921: 546–547)

This binary distinction is indeed belied by the facts: many spiritual writers are both ascetics and mystics. Even if asceticism and mysticism can carry antonymic semantic values, in mystical text it is possible to detect the presence of *complex terms.*

Derived from the elementary structure of signification, the complex term is defined by the “both … and” relation that the terms s1 and s2 of the axis of contradictories in the semiotic square contract, after preliminary syntactic operations (Greimas and Courtés 1982 [1979]: 47). As noted by the two authors:

The ‘coexistence of contraries’ is an arduous problem, inherited from a long philosophical and religious tradition. V. Brøndal introduced it into linguistics, recognizing the existence of complex terms in the articulation of the grammatical categories of certain natural languages. (Greimas and Courtés 1982 [1979]: 47)

In other terms, the presence of both mystical and ascetic values in religious writings can be considered a feature of this genre, and their artificial separation can lead to a misreading.

Furthermore, Weber’s views about the passivity of the mystic are contradicted by the political engagement of many charismatic personalities such as Hildegard of Bingen, Clare of Assisi, and Simone Weil; Catherine of Siena, Therese of Lisieux, and Marthe Robin assisted prisoners sentenced to death (Guitton 1985: 157–158); Padre Pio of Pietrelcina founded the hospital of San Giovanni Rotondo, and St. Catherine Fieschi managed the Prammatone hospital in Genoa. The relation between mysticism and social engagement is stable and well-known (De Certeau 1968–1975).

In this perspective, it is difficult to apply Weber’s distinction to concrete cases. It is, ultimately, a case of *semiotic ideology* (Keane 2018) applied to the domain of the religious discourse. Weber’s distinction is functional, serving to prove his theses about the dependence of economic development from cultural structures. Perhaps his views about Protestantism as a secularized asceticism disclose an insight on this religious culture; however, to this purpose, Weber reduces the meaning of religious practices to the attempt of reaching a state of grace, while every Christian ascetic knows that an omnipotent God cannot be forced to save anybody. Rather, many exemplary ascetics respond to a correlational hierarchy (i.e., *a class of classes*, according to Hjelmslev 1961 [1943]: 74) in which spirit, reason, and soul control matter, passion, and body. This hierarchy *produces* the meaning of the ascetic experience.

Furthermore, semiotic analysis lets a very different distinction emerge between ascetics and mysticism, consisting of their respective expression planes. In particular, mysticism connects experience to spiritual values through writing, while asceticism does not need writing since its expression plane is the body. Ascetics do not need to describe hunger or solitude, while mystics (or their popularizers) usually spend a lot of words to paradoxically describe the indescribability of the mystical union with God (De Certeau 1968–1975). Weber (1978 [1921]: 545) writes that “the unique character of mystical knowledge consists in the fact that, although it becomes more incommunicable the more it is specifically mystical, it is nevertheless recognized as knowledge.” He is victim of a rhetorical figure: without communication, mystic experience would remain an inconsistent private fact.

### 1.1 Padre Pio and Ludwig Wittgenstein

The case study consists of some excerpts from Padre Pio’s letters and from Ludwig Wittgenstein’s diaries. In both cases, mysticism allows the authors to develop a spiritual reading of their sufferings and anguish during World War I. They have been chosen, among others, because of their great differences. Padre Pio belongs to a very poor family of peasants from Pietrelcina, in the underdeveloped countryside of southern Italy. His father and his brother had to emigrate to pay his religious instruction. During World War I he was forced to serve despite his chronic bronchitis, and hospitalized.

Ludwig Wittgenstein belong to a very rich Viennese family of assimilated Jews. His mother was catholic, and young Ludwig’s private Sunday school teacher would later become a bishop (McGuinness 1988: 43).2Despite this catholic education, according to Gorlée (2020: 71–84) Wittgenstein’s use of a cypher code in his secret diaries reveals an unsuspected influence of kabbalistic tradition: a secret mystical faith. He had the best instruction that money could buy and volunteered as a simple soldier on the Russian front. Provided the difference between the two authors, the similarities between their spiritual researches cannot be explained through historical or sociological reasons. Both Wittgenstein and Padre Pio’s early mystical research was not influenced by tradition and by institutions, but by personal readings: Gemma Galgani inspired the future saint, while during the war the future philosopher read Tolstoy, Dostoevsky, Emerson, and Nietzsche. Their spiritual choices exemplify the *fourth secularization*, which followed the industrial age (Berzano 2019).3Originally introduced by Max Weber to explain the relationship between protestantism and capitalism, the notion of secularization has been further developed by Luigi Berzano. The author distinguishes different periods of secularization: the relationship described by Max Weber corresponds to the third one. The fourth one, triggered by the industrial revolution, is characterized by a “horizontal” religiosity, which borrows spiritual notions and practices from neighboring cultures and shows a weaker tie with its own tradition.


Wittgenstein and Padre Pio are good counterexamples to Weber’s distinction between mysticism and asceticism for different reasons. First, both mystic and ascetic themes are present in their writings.

Thus, the two *weltanschauungs* are compatible. In particular, as it will be shown below, Wittgenstein provides a clue to the opposition between spiritual activity and passivity: the mystic tries to escape the antonymic relation between them through a neutral term (“not to will and not to will not”). Second: mystical passivity does not prevent Padre Pio and Ludwig Wittgenstein to take sides in the war. Wittgenstein volunteered for military services and he was decorated. Padre Pio wrote a long letter about the meaning of duty in times of war:

All of us have duties, very serious duties to perform at this crucial moment, Raffaelina. Let us carry them out faithfully and preservingly … Let us accept courageously and cheerfully the orders which come from the top and let us do our duty in accordance with our state in life. (Da Pietrelcina 2002 [1975]: 457)

Of course, this ethical attitude did not prevent Wittgenstein and Padre Pio from describing and criticizing the worst aspects of the war in their writings. The nexus between performing one own’s duty and accepting one own’s state in life recalls such classical books as the *Bhagavad Gita*. During his mystical dialogue with Krishna, prince Arjuna asks about the purpose of renunciation and of the renounced order of life. In his answer, the Supreme Personality of Godhead distinguishes among different kinds of renunciation. Some of them, such as sacrifice, charity, and penance are not to be given up. However, prescribed duties should never be renounced: “By worship of the Lord, who is the source of all beings and who is all-pervading, a man can attain perfection through performing his own work” (Prabhupada 1968: 985).

It is better to engage in one’s own occupation, even though one may perform it imperfectly, than to accept another’s occupation and perform it perfectly. Duties prescribed according to one’s nature are never affected by sinful reactions. (Prabhupada 1968: 987)

According to this perspective, men should seek perfection in their own work, controlling the activities of the mind, life, and senses. Though this attitude seems very similar to Weber’s idea of an inner-worldly asceticism, in India it did not trigger the foundation of capitalism. It rather justified and conserved the caste system. The example is useful to illustrate the risks related to comparative studies about mysticism, seeking only similarities. Of course, Padre Pio’s intent was not to preserve the social order: rather, he was convinced that war was part of God’s plans in His wisdom.

### 1.2 Goals and structure of the argument

The paper will adopt a method of analysis inspired to Umberto Eco’s theory of sign production, presented in Section 2, to analyze a letter of Padre Pio’s to his spiritual daughter (Section 3) and some excerpts from Wittgenstein’s diaries (Section 4). Both the authors react to war and anguish by linking spiritual values to their bodily experience. They achieve this goal using metasemiotic statements introduced by speech acts: from the adopted point of view, sign production is a *labor*. The grid of analysis allows a comparison (Section 5). Mysticism appears as a semio-technique: the mystic actually produces the semantic values with which he wishes to join. By doing so, the mystic modifies her/his subjectivity, accepting pain, anguish, and the prospect of death. As a literary genre, mysticism shows also some “specificities”: semiotic labor is not directed toward system making, changing, and observing, but only toward code-making and the content-continuum.

## 2 Methods

**Figure 1:**
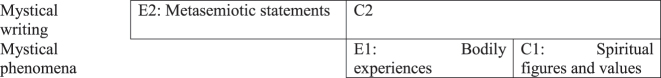
The relationship between mystical writing and phenomena can be represented as the relationship between metsemiotics and object-semiotics.

In what follows, I will reconstruct a fragment of Padre Pio’s and Ludwig Wittgenstein’s semantic universe by describing the form of Padre Pio’s mystical writing. In particular, we will search his letters for *metasemiotic statements* concerning a semiotic relation located in an object-semiotics whose expression plane is mainly composed of bodily experiences such as “pain,” “fear,” “consolations,” and whose content plane is mainly composed of spiritual figures and values associated to them, such as in the schema in Figure 1.

The analytic description of the metasemiotic statements will be made possible by Umberto Eco’s notion of *semiotic labor*.

### 2.1 Semiotic labor

According to Eco (1976 [1975]) when producing a new message, the sender can work on all the elements of the semiotic function: purport, form, relation between forms. In particular, according to theory of sign production, we can identify three kinds of semiotic labor:

– first, a labor focusing on either the purport or the form of the expression plane, aimed at the production of signals, the units whose combination will be used to produce a message.
– second, a labor focusing on either the purport or the form of the expression plane, aimed at relating the content of the message with the world, as far as the latter is referred to or presupposed by the message, its sender and receiver.
– third, a labor focused on the relationship between the form of the expression and the form of the content plane, to relate contents to signals. It is a work aimed to make, observe, change, or switch the code.

**Figure 2:**
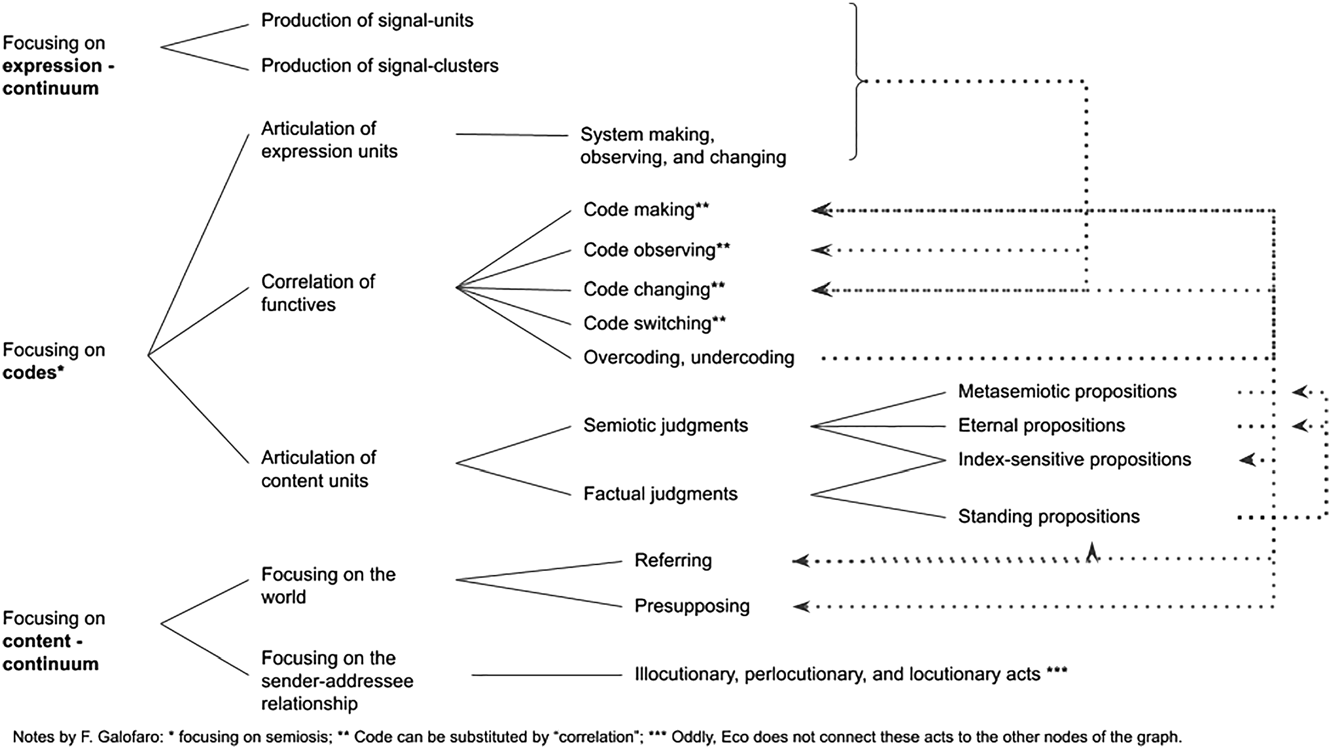
Kinds of semiotic labor. Source: Eco (1976 [1975]: 154).

The logical connections between each kind of labor are shown in Figure 2.

*Semiotic judgements* represent the kernel of Eco’s theory. They preside over the birth of meaning, or, at least, of the awareness of the significance. These judgements link the expression plane to the content plane: for example, “Bachelors are male”; “This is an exposition of a sacred object.” Generally speaking, they have the form “this means that.” Sometimes they are perlocutionary acts having an effect on the world. For example: “(From now on) this is my body which will be given up for you.”

The different kinds of semiotic labor listed in Figure 2 are logically interconnected: for example, “the moon has been walked on by human beings” is a *factual judgment* the first time it is pronounced, but it becomes a *metasemiotic judgement* when it is accepted by a society as true. Thus, many factual judgments can change the codes shared by society (*code changing*). In a similar way, the labor to check whether certain expressions refer or not to actual properties of the world and to interpret them (*referring, presupposing*) is strictly linked to *factual judgments.*

### 2.2 Semiotic labor between post-structuralism and pragmaticism

The development of a method of analysis starting from the *theory of sign production* may seem an exercise of retro-semiotics. However, according to Eco (1986 [1984]: 2, 80, 82, 117), global structures, codes, and even the labyrinthic encyclopedia are nothing but regulative hypotheses *in the service of a semiotics of sign production.* Thus, this chapter of Eco (1976 [1975]) was not considered outdated by the author:

This review of the possibilities of sign production shows that there is a semiosic continuum which goes from the strongest kind of coding to the most open and indeterminate. The task of a *general semiotics* is that of tracing a single formal structure which underlies all these phenomena, this structure being that of the inference which generates interpretation. (Eco 1986 [1984]: 38)

At the basis of the typology of semiotic labor (Figure 2), borrowed by the Marxist semiotic scholar Rossi-Landi (1968), there is a heteroclite epistemology. The principal subdivision into expression and content forms articulating two *continua* is a plain reference to Hjelmslev (1961 [1943]). Coherently with this framework, the connection between two forms is granted by metasemiotic statements. However, in the figure, classical topics in analytical philosophy and in pragmaticist semiotics such as reference and presuppositions are considered in reciprocal connection with metasemiotic statements, while Eco does not relate linguistic acts to them. The reason could be, in all likelihood, a doubt. Linguistic acts and metasemiotic statements coexist in ordinary speech; in religious, it is difficult to distinguish between them. Let us consider, for example, the following verse from the *Magnificat:*

Ὅτι ἐποίησέ μοι μεγαλεῖα ὁ δυνατὸς καὶ ἅγιον τὸ ὄνομα Αὐτοῦ

It is difficult to draw a boundary between the simple observation expressed through factual judgements (‘the Almighty has done great things for me, and his Name is holy’) and the illocutionary and perlocutionary effects well expressed by the Hebrew formulaic expression *Baruch Hashem,* corresponding to ἅγιον τὸ ὄνομα Αὐτοῦ. In line with Austin (1962), many locutionary acts hide illocutionary and perlocutionary effects under factual statements. Judgements and acts should be seen as two functions served by the same utterances.

Despite these uncertainties, Eco was convinced that Hjelmslev’s and Peirce’s epistemologies were complementary. For this reason, he was often criticized by both Peircean and Hjelmslevian scholars. However, there is at least a kernel topic that deserves thorough investigation: the notion of *presupposition.* As I will explain below, in Hjelmslev, *presuppositions* are central since they allow linguists to link the expression plane to the content plane by *catalysis*. In other terms, both semiosis and the content plane are inferred. *Presuppositions* are the reason of Eco’s interest toward pragmatics. Together with Patrizia Violi, he also proposed a formal language to analyze them (Eco and Violi 1987). Eco (1979) deepened this theme trying to complement Greimas’ generative trajectory with a description of the semiotic efforts made by the reader to actualize the content plane of the text, with the mediation of the circumstances of utterance and of culture.

### 2.3 The form of a metasemiotic statement

As will be shown below, mystical knowledge is acquired by selecting some elements of the mystic’s bodily experience as a plane manifesting mystical values. It is possible to identify:

– a labor of articulation of expression units, consisting of the observation of one’s sensations and their selection;
– a labor of judgments which articulates the form of the Content;
– a labor of correlation of functives, aimed to code making

The page is the workplace: using the verbal semiotic system the mystic produces metasemiotic statements aimed at the production of a second, nonverbal semiotic system {body: spirit}. Through writing, padre Pio concatenates different figures, both of the expression and of the content, creating and linking coherent planes.

The analysis will evidence semiotic subsets of three kinds: *elements, lists*, and *dictionaries*:4I borrow the notation system from the programming language Python, because of the need of univocity and recursiveness in the description of the form of the content of writing.

– Elements are *isotopies:* “the recurrence of semic categories, be they thematic (or abstract) or figurative” (Greimas and Courtés 1979: 164). Isotopies are semantic subsets belonging to the expression or to the content plane of the object-semiotics. In the present paper, these sets will not be further analyzed. They are labelled and surrounded in quotes as in the following examples: “Pain”; “Jesus hides.”
– Lists are *semantic subsets* that are chains of other semantic subsets, all on the same hierarchical level. Lists include elements, other lists, and dictionaries. Lists are shown in brackets, as in the following example: [“Caused by Satan,” “Jesus hides”]. Lists are commutative: the order of the elements is not relevant.
– Dictionaries represent *semiotic relations between subsets* of the expression and of the content plane. The semiotic relation, noted by colon, can connect elements, lists, and other dictionaries. Dictionaries are enclosed in curly brackets, such as in the example {“Pain”: “Jesus hides”}. In a dictionary, the order of the subsets is noncommutative: on the left, we mark the subsets belonging to the manifesting plane, on the right, the subsets belonging to the manifested plane.

The rules to describe metasemiotic statements will be presented in a formal, generative fashion, by deriving each metasemiotic statement from their general form through a finite series of substitutions. This formal method aims to reconcile Eco’s typological point of view on semiotic labor with “a procedural method by means of which a given text can be comprehended through a self-consistent and exhaustive description” (Hjelmslev 1961 [1943]: 16).

2.1) E: C is a semiotics

2.2) “x => y” means “the letter x can be replaced by the letter y”

2.2) E, C => {E: C}

2.3) E, C => [i_1_, i_2_ … i_n_]

2.4) x => A, B, … Z

2.5) i_X_ => “isotopy”

– Rule 2.1 shows the general form of a semiotics. Metasemiotic statement: letter E represents the expression plane of a statement; letter C represents the content plane.
– Rule 2.2 introduces the sign of substitution. Any substitution can be applied, recursively, on each component of the metasemiotic statement.
– According to rule 2.3, both the expression and the content plane of a semiotics can be substituted by a semiotics. This rule allows for the formation of metasemiotics and connotative semiotics, i.e., “a semiotics one or more of whose plans is (are) a semiotics” (Hjelmslev 1961 [1943]: 138).5In our corpus, the considered metasemiotic statements are – most likely – part of a connotative semiotics. However, this point seems not relevant to the research question. For example, it is possible to generate E: {E: C}.
– According to rule 2.4, any of the two symbols E and C can be substituted by a list. This way it is possible to generate, for example, E: {[i_1_]: [i_2_, i_3_]}.
– Rule 2.5 allows for the substitution of every symbol with a capital letter. This provides us a way to identify the metasemiotic statements, for example: A: {[i_1_]: [i_2_, i_3_]}. It is also possible to derive, recursively, metasemiotic statements about other metasemiotic statement, such as A: {[i_1_]: [i_2_, B]}.
– Finally, according to rule 2.6, any symbol i_x_ can be substituted by a label representing an isotopy, for example: A: {[“pain”]: [“Jesus hides,” B]}. This rule can be puzzling, since the elements of the expression plane of the object-semiotics often represents bodily experiences; however, one should remember that bodily experiences can always be considered as elements of the content plane of a metasemiotic statement about an object-semiotics.

The following examples can be derived from the form (2.1) through a recursive application of the rules:

A: {[“pain,” “spirit,” “darkness,” “brightness”]: [“caused by God”]}

B: {[“dirt,” “deformity”]: A}

The first example can be read: the metasemiotic statement “A” expresses the following content: the isotopies “pain,” “spirit,” “darkness,” and “brightness” manifest the isotopy “caused by God.” As for the second example, the metasemiotic statement “B” expresses the following content: the isotopies “dirt,” “deformity” manifest the metasemiotic statement A.

## 3 Padre Pio’s letter

The first example of mystical writing is a letter that Padre Pio addressed to his first spiritual daughter Raffaelina Cerese during World War I. Raffaelina was a 47-year-old aristocratic woman of Foggia. Padre Pio never met her in person during the period of the spiritual direction. To stay in touch, they exchanged letters, and soon developed a close friendship. At that time, Padre Pio was a 28-year-old young friar. He had not received the stigmata; the mystical phenomena he was into were known only to his spiritual directors. Padre Pio had been called up to the army and sent to the military hospital in Naples for suspected tuberculosis.

Pietrelcina, 5 December 1914

Beloved daughter of the heavenly Father

May the grace of the divine Spirit always reign supreme in your soul and may it guide you to greater Christian perfection. Amen.

I refrain from telling you about my situation, both because I lack the strength to do it and because you have already heard all from our very dear Padre Agostino. Tomorrow, 6 December, I have to leave for Naples as I have been assigned to the Tenth Medical Corps.

My daughter, I cannot stand up to this very harsh trial to which I am being subjected. By a pure miracle I have been sustained up to this moment, but for the future? Alas, I can no longer lay claim to this! I yearn for the moment when I shall be released from this extremely dark prison, and may it be as soon as possible.

All I ask from the divine mercy of the Lord is that He may not allow His servant to depart for a better land with this two-edged sword piercing his heart, by which I mean my twofold exile.

Together with all those who are united with me in the bond of Christ’s charity, my daughter, keep me company by asking insistently for what I myself am asking. In the meantime I want you and good Francesca, Annita and all the others who are dear to me to start at once for me the three novenas to the Virgin of Pompei, by reciting daily the entire Rosary and by frequent Communion, which I earnestly hope will be every morning.

Thank your beloved sister on my behalf for the news she sent me. I appreciate immensely the picture of your own beautiful Virgin which you sent me. May Jesus reward you.

I end here, my dear Raffaelina, because I can go on no longer. May Jesus always console you. (Da Pietrelcina 2002 [1975]: 549–550)

The letter expresses both physical and moral sufferance. Padre Pio’s reacts by assigning a spiritual meaning to them.

### 3.1 Metasemiotic propositions

The letter presents different metasemiotic propositions acting on the correlation between functives of the expression and the content plane of the object-semiotics, consisting of experiences and spiritual meanings, respectively. For example:

Tomorrow, 6 December, I have to leave for Naples as I have been assigned to the Tenth Medical Corps. My daughter, I cannot stand up to this very harsh trial to which I am being subjected. By a pure miracle I have been sustained up to this moment, but for the future?

The statement can be analyzed as follows:

A: [“enlistment”]: [“trial,” “weakness”]}

The text returns to metasemiotic statements assigning further meanings to them:

… I shall be released from this extremely dark prison, and may it be as soon as possible.

Thus, the trial is seen as a dark prison, and death represents the liberation.

B: {[A]: [“Dark prison”]}

C: {[“Death”]: [“liberation from,” B]}

### 3.2 Metasemiotic propositions and presuppositions

Unfortunately for him, Padre Pio is aware that his death would represent a failure of the trial:

… He may not allow His servant to depart for a better land with this two-edged sword piercing his heart, by which I mean my twofold exile.

In order to understand the “twofold exile” the reader must know, as Raffaelina did, that in that period Padre Pio was forced to quit the convent and to return home due to his illness. To be manifested, the elements of the content plane presuppose the “absence from the convent” alongside the dark prison.

D: {[B, “absence from the convent”]: [“two-edged sword,” “twofold exile,” “pain”]}

E: {[C: “may not be allowed by God until,” D]}

### 3.3 Labor focused on the relation between sender and receiver

The letter presents different illocutionary and perlocutionary acts:

– May the grace of the divine Spirit always reign supreme in your soul and may it guide you to greater Christian perfection. Amen.

– May Jesus reward you.

– May Jesus always console you.

These formulaic blessings are connected to the general isotopy of the “path to perfection” and with the thematic role of the spiritual guide embodied by Padre Pio. Furthermore, there are prayers and requests of prayers:

– Alas, I can no longer lay claim to this! I yearn for the moment when C

– All I ask from the divine mercy of the Lord is that’ E

– Keep me company by asking insistently for E

**Figure 3:**
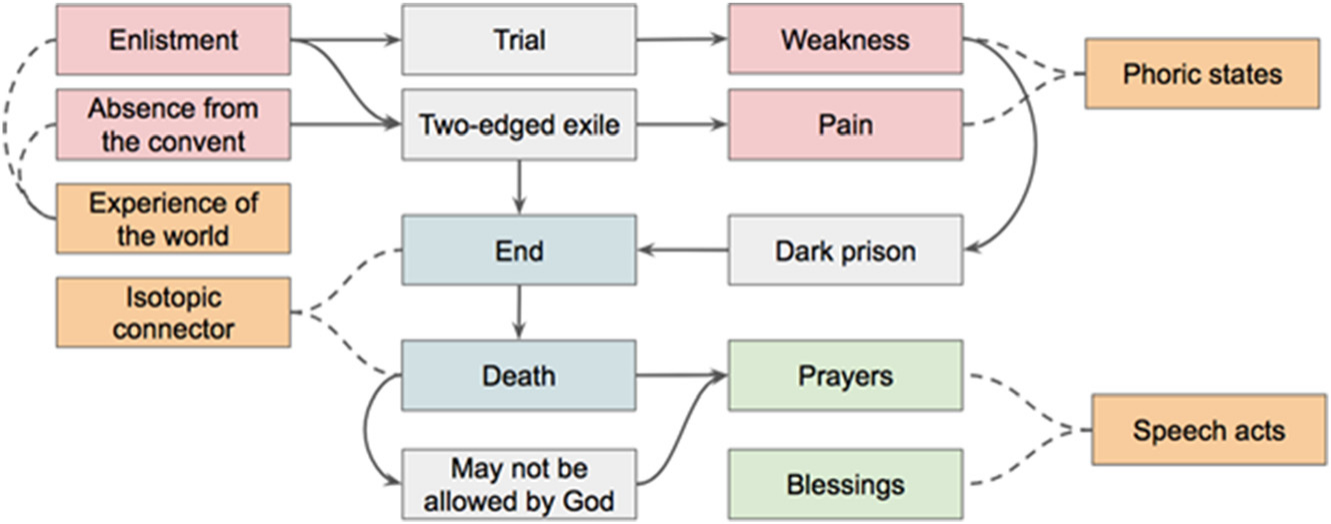
The form of the spiritual content of Padre Pio’s letter.

Each metasemiotic statement is introduced by a speech act. The overall structure of the letter is represented in Figure 3.

Figure 3 shows how metasemiotic statements, introduced by speech acts (prayers), recursively connect phoric states (weakness, pain) to the experience of the word (enlistment, absence from the convent), giving birth to one or more spiritual isotopies (trial, two-edged exile, dark prison) and isotopic connectors (end, death).

## 4 Wittgenstein’s war diaries

During World War I, Ludwig Wittgenstein was enrolled as a volunteer in the Austro-Hungarian Army and sent to the Eastern front. He left Cambridge, where he was a university student and a pupil of Bertrand Russell and George Moore. During the war, he kept a diary, recording his logical research in a notebook (Wittgenstein 1961). He also noted his everyday experience: since these paragraphs are encrypted, they were published thirty years later in plaintext (Wittgenstein 1991), against the will of the curator of Wittgenstein’s work, G. H. von Wright, who had judged them void of any scientific value. The analysis combines entries of two parts of the diaries to frame Wittgenstein’s research in its historic circumstances.

### 4.1 During the Brusilov offence

During the war, Wittgenstein writes passages filled with anguish and hope, expressing the expectation of death.

May 16th 1916 (CODED) In third position. As usual, much effort. But a great grace too. I am weak as always! I cannot work. Today I sleep under the infantry fire, and will probably perish. The Lord be with me! Forever. Amen. I am a weak man, but He spared me so far. Amen. Blessed the Lord forever. I offer my Soul to the Lord. (Wittgenstein 1961)

On June 4th, 1916, the Brusilov offensive started, one of the most lethal attacks of the history of wars, which ended on August 15th. Wittgenstein was involved in heavy fighting, and he was eventually decorated (Preston 2020; Waugh 2008: 114). What follows is a selection of the entries from July 5th and July 30th. Despite the offensive, Wittgenstein worked hard on his research, in the fear of not being able to complete it. Many uncoded passages will be reported in his *Tractatus Logico-Philosophicus* (Wittgenstein 2002 [1921]). The majority of them will be inserted in Section 6.4, focusing on the limits of the world and what Wittgenstein calls *das Mystiche* (‘the mystical’). One of the *coded* passages, dated July 7th, echoes the famous Section 7: “What we cannot speak about we must pass over in silence.” A precise analysis of the influence between Wittgenstein’s religious passages and his scientific work on the relation between language and world is outside the scope of the present paper (on this point, see Galofaro 2022, 2023; McGuinness 1988: 245). The present, short selection is aimed to underline the relation between mystical writing and anguish.

July 6^th^, 1916 (UNCODED) And in this sense Dostoevsky is right when he says that the man who is happy is fulfilling the purpose of existence …

July 6^th^, 1916 (CODED) Colossal exertions in the last month. I reflected at length on every possible thing, but oddly enough I cannot establish the connection with my mathematical modes of thought

July 7^th^, 1916 (CODED) But the connection will be established! What cannot be said, *cannot* be said!

July 8^th^, 1916 (UNCODED) … When my conscience upsets my equilibrium, then I am not in agreement with Something. But what is this? Is it the world?

Certainly it is correct to say: Conscience is the voice of God

July 9^th^, 1916 (CODED) People are grey villains. And yet you must not annoy yourself about them. Their words must not invade you. If they do not address you, it is still easy to stay calm. But if they are cheeky and rude towards you, it wells up inside you. Do not be upset. Fretting benefits you nothing.

July 19^th^, 1916 (CODED) It’s still annoying me. I’m a weak man

July 20^th^, 1916 (CODED) Just keep working so you’ll become a good person

July 29^th^, 1916 (UNCODED) … Is it possible to will good, to will evil, and not to will?

Or is only he happy who does not will?

“To love one’s neighbor” would mean to will! (Wittgenstein 1961)

In this period, Wittgenstein considered himself in exile between poor specimen of men in terrible circumstances, in order to be purified. He reflected on the meaning of sin considered as a false conception of life. He concluded that he could not lead an authentic life if he had fallen prey of the passions. He was convinced that the Spirit would help him to cope with the terrible condition of the war, and considered happy periods, when he was allowed to work, as a “grace.”

### 4.2 Metasemiotic and eternal propositions

The analysis will start from the individuation of simple metasemiotic propositions, connecting experience and values. The first is a reflection of Dostoevsky’s ethics:

And in this sense Dostoevsky is right when he says that the man who is happy is fulfilling the purpose of existence.

A: {[“happy life”]: [“doing the will of God”]}

This crucial passage presents a theme of Wittgenstein’s mystical concerns: the relation between happiness and ethical actions. According to Wittgenstein, this nexus affects the subject’s point of view on the world. Wittgenstein refers to *The Brothers Karamazov*. In part I, book 2, chapter 4, Father Zosima is speaking to a mother, presented by the author as a “lady of little faith,” who cannot be happier since she is convinced that the elder monk has cured her young daughter. The lady asks after the old man’s health. Father Zosima knows that his days are numbered, and he answers:

I am extraordinarily better today. But I know that it’s only for a moment. I understand my disease now thoroughly. If I seem so happy to you, you could never say anything that would please me so much. For men are made for happiness, and anyone who is completely happy has a right to say to himself, ‘I am doing God’s will on earth.’ All the righteous, all the saints, all the holy martyrs were happy. (Dostoevsky 2017: 64)

The passage impressed Wittgenstein, who was expecting to die at any moment. The link between happiness and ethics became central in Wittgenstein’s concerns. In turn, unhappy life seems to depend on conscience:

When my conscience upsets my equilibrium, then I am not in agreement with Something. But what is this? Is it the world?

Certainly it is correct to say: Conscience is the voice of God.

B: {[“Conscience”]: [“voice of God”]}

C: {[“unhappy life”]: B}

In order to agree with the will of God, Wittgenstein tries to suppress his own will. This way he re-interprets a classical theme of mystic speculation: passivity and detachment:

It’s still annoying me. I’m a weak man

D: {[“rage”]: [“weakness”]}

Just keep working so you’ll become a good person.

E: {[“work”]: [“good”]}

By achieving a passive state, the mystic philosopher is in agreement with a different will, upon which he is dependent, identified by Wittgenstein with “the world” and with God. Thus, by reaching a passive state, he is doing the will of God. However,

Is it possible to will good, to will evil, and not to will?

Or is only he happy who does not will?

“To love one’s neighbor” would mean to will!

Thus, it is possible to describe this position as follows:

F: {“happy life”: [“to love,” “not to want”]}

Wittgenstein feels an implicit contradiction between “To love one’s neighbour” and “not to will.” He is trying to escape the category of will, constructing a neutral term: “not to do and not to do not.” According to Marsciani (1988), an interesting function of neutral terms is to *escape the category.* By reaching this kind of passive state the mystic is capable of achieving what she/he desires: a direct contact with the divine, or knowledge related to it: precisely the mystic knowledge of the final part of Wittgenstein’s *Tractatus*. All considered, the ascetic point of view on life can be described as follows:

G: {“detachment,” “asceticism”: F}

### 4.3 Labor focused on the relation between sender and receiver

As in Padre Pio’s case, the diaries present many speech acts:

In third position. As usual, much effort. But a great grace too. I am weak as always! I cannot work. Today I sleep under the infantry fire, and will probably perish. The Lord be with me! Forever. Amen. I am a weak man, but He spared me so far. Amen. Blessed the Lord forever. I offer my Soul to the Lord

These invocations are connected to general isotopies such as “weakness,” “grace,” “work,” and with the thematic role of the merciful embodied by God. Furthermore, Wittgenstein addresses to himself some admonitions:

People are grey villains. And yet you must not annoy yourself about them. Their words must not invade you. If they do not address you, it is still easy to stay calm. But if they are cheeky and rude towards you, it wells up inside you. Do not be upset. Fretting benefits you nothing.

**Figure 4:**
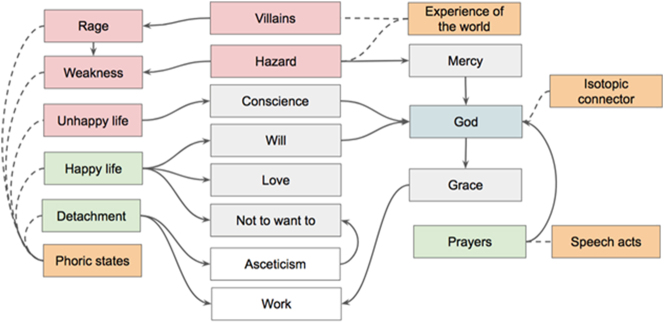
The form of the spiritual content of Wittgenstein’s entries.

In this case the association regards such isotopies as “rage,” “detachment,” “asceticism,” “weakness,” and the thematic role of the villains. The overall structure of the passage is represented in Figure 4.

As in Padre Pio’s letters, metasemiotic statements, introduced by speech acts (invocations, admonitions), recursively connect phoric states (weakness, rage, happy/unhappy life, detachment) to the experience of the word (villains, hazard), giving birth spiritual isotopies (Conscience, Will, Love, Not want to, Asceticism, Work, Grace, Mercy) and isotopic connectors (God).

## 5 Comparison

Considered as a *form of life* (Fontanille 2015), mysticism allows the subject to survive in the hostile war environment, to preserve one’s form from other forms (enemies, comrades, senior officers) and to cope with an ever-possible sudden death. Despite of many differences between Wittgenstein’s and Padre Pio’s language, culture, education, and social background, their mystical reading of the war is similar. Their research can be considered as a syntagmatic process, with similar stages: asceticism and passivity represent the reaction to a painful experience that they cannot avoid without the active intervention of the Spirit or the Grace. Provided the great difference between the two authors, the similarities between their spiritual research cannot be explained by historical or sociological reasons. The explanation is mainly structural: in both cases, the mystic discourse represents a transformation of the ordinary relation between the subject and the objects of the world. “What is transformed in mystical discourse is the modality of the relation: objects are there, but the value of their values is challenged, the relation between subject and object is well-posed, but what it is worth?” (Panier 1986: 27). To cope with the devaluation of the world, caused by the exceptional circumstances represented by war, Wittgenstein and Padre Pio associate a spiritual reading to bodily experiences such as pain, weakness, anguish of death. The association needs writing as a metalinguistic laboratory: Padre Pio’s and Ludwig Wittgenstein’s empirical explorations can be described by a theory of sign production as a set of metasemiotic asserts connecting the expression and the content plane.

### 5.1 The role of catalysis

*Catalysi*s is the kernel of semiotic labor*:*

The possibility must be foreseen that the registration of certain functions may, by virtue of solidarity between function and functive, oblige us to interpolate certain functives which would in no other way be accessible to knowledge. (Hjelmslev 1961 [1943]: 94)

The two aspirant mystics fill the gaps in the content plane of their experience by interpolating some missing spiritual values. Of course, in the quoted passage, Hjelmslev describes the construction of a *scientific* semiotics; however, there is little doubt that every semiotic labor aiming to link the expression and the content plane needs catalysis, being this the very condition of possibility of semiosis:

Linguistic theory prescribes a textual analysis, which leads us to recognize a linguistic form behind the “substance” immediately accessible to observation by the senses, and behind the text a language (system) … The kernel of this procedure is a catalysis through which the form is encatalyzed to the substance, and the language encatalyzed to the text. (Hjelmslev 1961 [1943]: 97)

### 5.2 Speech acts

For similar reasons, the mystic semiotic labor needs a speech act to become effective. It is represented by prayers, blessings, invocations, and admonitions. We can represent this fact with the symbol ⊢, meaning “let.” Consequently, the general structure used to introduce metasemiotic statements is:

⊢ E: {[i_1_, i_2_ … i_n_]: [i_1_ , i_2_ … i_n_]}

In fact, the mystic can assign a spiritual reading to the word of experience only with the Grace of Lord and in communion with the divinity. Speech acts are *constants*, selecting different spiritual isotopies as *variables* (Hjelmslev 1961 [1943]). The presence of linguistic acts reveals how mystic readings of reality are conventional; however, the validity of the convention is ensured by the divinity, praised by the mystic.

### 5.3 Semio-technique and subjectivity

The great similarities between Padre Pio’s and Ludwig Wittgenstein’s semiotic labor allow to consider mysticism as a semio-technique (Galofaro 2017). Though the mystic aims to contemplation, this state is reached through a work:

Try to forget all created things that he ever made, and the purpose behind them, so that your thought and longing do not turn or reach out to them either in general or in particular. Let them go, and pay no attention to them. It is the work of the soul that pleases God most. (Wolters 1978: 61)

By applying a semio-technique, the mystic actually produces the semantic values with which he wishes to join. The mystic praises God to reach a detached state, featured by spiritual values; by doing so, he produces this state and joins with these values. Writing, prayer, meditation, and contemplation allow the mystic to operate on her/his own subjectivity.

### 5.4 System-making and subjectivity

The metasemiotic statements that can be found in mystic literature aim to code-making. According to Umberto Eco, a different semiotic labor is aimed to *system-making* (Figure 2). This work is directed toward the units of the expression plane and is absent in the examples of mystic writing presented above. One explanation is that the mystic has no control over the expression plane, i.e., on the bodily experiences that he considers symptoms of the divine. He passively submits to “pain” and “joy,” to “anguish” and “consolation.” Thus, the mystic can only work on code-making. As a consequence, the mystic experiences the illocutionary force exerted by the divine and its perlocutionary effect on the body, reduced to a mere material support for the divine expression (De Certeau 1968–1975). From this point of view, the subject is not an a-priori condition of possibility of mystic experience, being rather its product. Mystic knowledge is “knowledge of the divine,” in which expression the genitive is both objective and subjective.

This explains the annihilation of the mystic as a subject of the enunciation: according to De Certeau (1987), it is a way to “let be” the Other, reproducing the relation Subject/Other at the level of the enunciation of the mystical discourse. The annihilation of subjectivity is one of the outcomes made possible by semiotic labor. Subjectivity symmetrically appears as an effect created by the text when system-making and system-changing prevail on code-making and on other kind of labor.

## 6 Conclusions

Mystics take a stand in conflicts. Weber remembers St. Bernard’s “mariolatry” and mystical contemplativeness (Weber 1978 [1921]: 537, 552) but forgets his active role in the promotion of the second crusade. Likewise, both Padre Pio and Wittgenstein associated a spiritual meaning to the war and coherently suffered its consequences. Weber's opposition between active asceticism and passive mysticism is not reflected in actual texts and shows the limits of his method, aimed to the construction of abstract ideal types, revealing the author’s semiotic ideology.

Furthermore, Weber’s characterization of mysticism as the attempt to reach a passive state seems unsatisfactory, since it does not take into account the *paradoxicality* of mystical discourse:

The active life has two parts, a higher and a lower, and likewise the contemplative has two parts too, a lower and a higher. These two ways of life are linked, and though they are different, each is dependent on the other. For what we call the higher part of the active life is the same as the lower part of the contemplative. A man cannot be fully active except he be partly contemplative; nor fully contemplative (at least on earth) without being partly active. (Wolters 1978: 71)

To produce the ideal type of the mystic, Weber narcotizes the paradoxical features of mystic discourse by selecting only the ones which is more functional to his thesis. It is a case of ideological *dispositio* 6It appears that Umberto Eco borrowed the term *dispositio* from rhetoric, where it has a different meaning, to focus his analysis of ideology on the organization of arguments. (Eco (1976 [1975]: 294–295). If the ideal mystic ever existed, paradoxicality would be one of her/his distinguishing marks. The acquisition of knowledge from passivity is obviously paradoxical. In order to do nothing, the mystic must do a lot of things: to pray, to praise God, to meditate, and so on. In our perspective, this is possible because the mystical discourse is a semiotic labor, involving intense fatigue:

WORK hard at it, therefore, and with all speed; hammer away at this high cloud of unknowing – and take your rest later! It is hard work and no mistake for the would-be contemplative; very hard work indeed, unless it is made easier by a special grace of God, or by the fact that one has got used to it over a long period. (Wolters 1978: 94)

According to the structural explanation hereby adopted, in fact, spiritual values are associated to the manifesting plane of the body by ascetic practices, such as continence, fasting, vigils, solitude. The ascetics has no need to describe the pangs of hunger, the blows of sleep, the details of temptations.

On the contrary, the mystics needs to associate spiritual values to experience through enunciation. To prove this, the paper provided examples of the semiotic labor aimed to produce a semantic reading of the mystic’s experience. In our examples, the mystics associate spiritual values to pain, anguish, fear through catalysis, interpreting them as divine trials. This is done thanks to metasemiotic asserts introduced and validated by speech acts: a semio-technique, producing the semantic values with which the subject wishes to join.

For these reasons, an important difference between mysticism and asceticism concerns their literary genre. While the first produces literature, plenty of amazing visions, sensations, and voices, the second produces manuals rich of spiritual advices addressed to the ascetics’ brothers and sisters. Treatises on mysticism are comparatively rarer, even though they are indisputable classics such as *The Cloud of Unknowing* or St. John of the Cross’ *Dark Night*. Scarce are the manuals addressed to the spiritual direction of mystics, such as Giovanni Battista Scaramelli’s *Direttorio mistico* (1754). In Eastern Christianity, a similar problem concerns hesychasm: the first presentation of a method dates to Nikephoros the Monk (thirteenth century), while the practice of the inner prayer is testified from the fourth century. Probably, this is because mystic work is mainly individual research. However, despite Max Weber’s efforts to pit them against one another, asceticism and mysticism coexist in religious discourse as they undoubtedly do in the fragments analyzed above.
